# Metformin Attenuates Angiotensin II-Induced Cardiac Inflammaging-Like Injury Through Coordinated Nrf2 Activation and NF-κB Suppression

**DOI:** 10.5812/ijpr-170947

**Published:** 2026-05-20

**Authors:** Huarui Li, Deming Fu, Umar Saeed, Zahra Zahid Piracha, Jianjun Bao

**Affiliations:** 1 Department of Geriatric, Xi'an Gaoxin Hospital, Xi'an, China; 2 Geriatric Comprehensive Care Center，TaiKang Yueyuan Hospital Guangzhou，Huangpu District，Guangzhou, China; 3 Department of Cardiovascular Medicine, Taiyuan Central Hospital, Peking University First Hospital, Taiyuan Hospital, Taiyuan, China; 4 Korea University College of Health Sciences, Seoul, South Korea; 5 Széchenyi István University, Győr, Hungary; 6 International Center of Medical Sciences Research (ICMSR), Islamabad, Pakistan; 7 Department of Cardiologyinternal, Xi’an TCM Hospital of Encephalopathy, Xi'an, China

**Keywords:** Metformin, Angiotensin II, Oxidative Stress, Nuclear factor-κB, Nuclear Factor Erythroid 2-related Factor 2, Cardiomyocyte Injury, Inflammaging-like, Senescence, Apoptosis, Wound Repair

## Abstract

**Background:**

Age-related cardiomyocyte vulnerability is driven by a convergent triad of persistent oxidative stress, chronic low-grade inflammation, and senescence-like signaling, which together accelerate functional decline and maladaptive remodeling. Angiotensin II (Ang II), a clinically relevant stress effector, amplifies reactive oxygen species (ROS) production and inflammatory activation, thereby impairing cardiomyocyte survival and repair capacity. Although metformin has emerging cardiovascular benefits beyond glycemic control, its coordinated capacity to counteract Ang II-driven inflammaging-like injury through the coupled regulation of antioxidant and anti-inflammatory pathways in human cardiomyocytes remains insufficiently defined.

**Objectives:**

This study aimed to determine whether metformin attenuates Ang II-induced injury and impaired repair in human AC16 cardiomyocytes and to evaluate whether this protection is associated with coordinated activation of Nuclear Factor Erythroid 2-Related Factor 2 (Nrf2) and suppression of Nuclear Factor-κB (NF-κB).

**Methods:**

Human AC16 cardiomyocytes were preconditioned with metformin (0.5 or 1.0 mM) for 2 h and then challenged with Ang II (0.1 - 2.0 μM) under continuous metformin exposure. The primary efficacy endpoints were cell survival, quantified using the MTT assay, and repair competence, assessed by scratch wound closure. Secondary mechanistic endpoints included inflammatory mediators (TNFA, IL6, and IL1B); senescence-associated markers (CDKN2A/p16 and CDKN1A/p21); antioxidant genes linked to Nrf2 signaling (NFE2L2/Nrf2, HMOX1/HO-1, and NQO1); NF-κB pathway activation; antioxidant and apoptosis-related proteins; Nrf2 compartmentalization; and intracellular ROS.

**Results:**

Angiotensin II induced a dose-dependent injury phenotype in AC16 cardiomyocytes, reducing viability from 100.0 ± 1.1% in control cells to 64.9 ± 1.6% at 1.0 μM and 53.0 ± 1.6% at 2.0 μM, while markedly impairing wound closure (80.0 ± 2.0% in control vs 32.3 ± 2.5% with Ang II). Metformin attenuated this injury in a concentration-dependent manner, restoring viability to 81.7 ± 1.0% with 0.5 mM and 89.7 ± 1.0% with 1.0 mM and improving wound closure to 52.3 ± 2.5% and 70.0 ± 2.0%, respectively. Angiotensin II also robustly increased inflammatory cytokine expression, with TNFA, IL6, and IL1B increasing to 27.86 ± 2.79-fold, 29.86 ± 2.99-fold, and 29.86 ± 2.99-fold, respectively, accompanied by NF-κB activation, ROS accumulation (187.7 ± 2.5% of control), apoptosis-associated signaling, and upregulation of the senescence-like markers p16 and p21. Metformin markedly suppressed these responses, reducing cytokine expression toward near-baseline levels, lowering ROS to 120.0 ± 2.0%, decreasing the BAX/BCL-2 ratio from 2.98 to 1.19, and reducing cleaved caspase-3 and cleaved PARP to 120% and 112% of control, respectively. In parallel, metformin enhanced Nrf2-associated antioxidant signaling, increasing NFE2L2 to 2.14 ± 0.17-fold, HMOX1 to 4.29 ± 0.30-fold, and NQO1 to 3.81 ± 0.27-fold, consistent with enhanced antioxidant defense under Ang II stress.

**Conclusions:**

Metformin attenuated Ang II-driven cardiac inflammaging-like injury and impaired repair, in association with reduced inflammatory signaling and enhanced activation of antioxidant pathways. These findings support a mechanistically coherent model in which NF-κB suppression and Nrf2 activation may contribute to metformin-mediated protection; however, direct pathway dependence requires confirmation through perturbation-based studies.

## 1. Background

Cardiovascular aging is increasingly recognized as a global clinical challenge driven not only by structural remodeling but also by the progressive loss of cardiomyocyte resilience. With advancing age, cardiomyocytes exhibit reduced survival, impaired reparative responses, persistent oxidative damage, and chronic low-grade inflammation that converges with senescence-like signaling to accelerate functional decline and predispose to heart failure. These interrelated processes create a self-reinforcing vulnerability phenotype in which stress responses become maladaptive, repair becomes inefficient, and injury thresholds are lowered. These features are consistent with an “inflammaging” biology that is highly relevant to the aging myocardium (1, 2).

Among the upstream stress systems that shape this phenotype, chronic neurohormonal activation is a key pathological driver, and Ang II represents a central effector molecule linking hypertension, cardiac stress burden, and myocardial injury. Angiotensin II promotes ROS generation through mitochondrial dysfunction and NADPH oxidase activation, initiating redox imbalance that directly weakens cardiomyocyte survival capacity and indirectly amplifies inflammatory signaling and apoptotic execution pathways (3-5). Beyond acute toxicity, sustained Ang II exposure fosters a repair-deficient state in cardiomyocytes, supporting the concept that cardiovascular aging is characterized not only by increased injury but also by reduced recovery and diminished regenerative competence.

At the molecular level, NF-κB functions as a pivotal bridge between oxidative stress and chronic inflammation in Ang II-driven injury. Reactive oxygen species-dependent activation of NF-κB enhances the transcription of pro-inflammatory mediators, including tumor necrosis factor-α, interleukin-6, and interleukin-1β, reinforcing senescence-associated secretory phenotype-like outputs that intensify inflammatory stress and accelerate cellular aging (6). In parallel, Ang II is known to induce the cyclin-dependent kinase inhibitors p16 and p21, key regulators of senescence-like growth-arrest programs that constrain repair responses and further compromise cardiomyocyte adaptability under chronic stress (7). Collectively, these pathways support an Ang II-driven inflammaging-like and repair-failure axis.

Consistent with this framework, a growing body of work across stress-injury models indicates that redox imbalance and inflammation are not independent injury features but tightly coupled drivers of endothelial dysfunction, tissue damage progression, and stress-responsive molecular remodeling (7-11). These studies highlight stress-linked biomarkers and redox-mediated cascades as actionable contributors to disease propagation and support the premise that pharmacological strategies capable of simultaneously dampening inflammation and restoring antioxidant control may offer protective potential in experimental cardiomyocyte injury settings (12, 13). However, preclinical progress is limited by the need for pharmacological approaches that act at the network level, resetting coupled inflammatory and antioxidant pathways rather than targeting isolated downstream endpoints.

Within this context, metformin is of particular interest as a widely available drug with accumulating evidence of cardiovascular benefits that extend beyond glucose lowering (14, 15). Metformin has been reported to improve mitochondrial function, reduce ROS burden, and suppress pro-inflammatory signaling in diverse cardiovascular disease models (16). Notably, attenuation of NF-κB activation and reduced inflammatory cytokine output have been described as key anti-inflammatory effects of metformin, aligning it with mechanisms relevant to inflammaging-associated injury. Importantly, metformin is also linked to activation of Nrf2, a master transcriptional regulator of cellular antioxidant and detoxification programs. Nuclear Factor Erythroid 2-Related Factor 2 controls the expression of enzymes such as heme oxygenase-1 and NAD(P)H quinone dehydrogenase 1, which collectively counteract oxidative stress and stabilize cellular homeostasis (1, 2). Dysregulation of Nrf2 signaling has been associated with heightened oxidative vulnerability in cardiovascular aging, whereas pharmacological restoration of this pathway confers protection against stress-induced inflammation, apoptosis, and tissue injury (1, 2). These observations position metformin as a plausible dual-axis regulator capable of suppressing inflammatory amplification while reprogramming antioxidant defenses.

Despite this emerging rationale, the integrated mechanisms by which metformin counteracts Ang II-driven inflammaging-like injury in human cardiomyocytes remain incompletely defined. Specifically, whether metformin can reverse the coupled phenotype of reduced survival, impaired repair, enhanced ROS generation, inflammatory cytokine induction, senescence marker upregulation, and apoptotic signaling under Ang II stress, and whether these effects are associated with coordinated NF-κB suppression alongside Nrf2 pathway activation, have not been comprehensively characterized in a unified experimental framework.

## 2. Objectives

The present study established a reproducible Ang II-induced injury and repair-failure model in human AC16 cardiomyocytes and prospectively evaluated metformin using predefined functional efficacy endpoints, namely, cell survival and reparative behavior, together with mechanistic analyses of inflammatory activation, oxidative stress, antioxidant signaling, apoptosis, and exploratory senescence-associated stress markers. This work was designed as a preclinical, cell line-based investigation intended to generate mechanistic insight rather than to support direct translational inference regarding the biology of the aging myocardium or cardiovascular therapy.

## 3. Methods

### 3.1. Study Objectives and Outcome Definitions

This study was prospectively designed to determine whether metformin functionally protects AC16 cardiomyocytes against Ang II-induced injury and to characterize the associated changes in inflammatory and antioxidant signaling. The primary efficacy endpoints were cell viability, measured using the MTT assay, and repair-related behavior, assessed by scratch wound closure. These outcomes were designated as the principal functional readouts because they directly reflect injury severity and recovery capacity under Ang II stress.

Secondary mechanistic endpoints included inflammatory transcriptional responses (TNFA, IL6, and IL1B), NF-κB pathway activation assessed by p65 phosphorylation and IκBα abundance, intracellular ROS generation, antioxidant-response gene and protein expression (NFE2L2/Nrf2, HMOX1/HO-1, and NQO1), Nrf2 nuclear enrichment, and apoptosis-related protein markers (BAX, BCL-2, cleaved caspase-3, and cleaved PARP). These outcomes were selected to test the mechanistically plausible hypothesis that metformin-associated protection is accompanied by suppression of inflammatory signaling and enhancement of antioxidant defense pathways.

Exploratory outcomes included senescence-associated transcriptional markers, specifically CDKN2A (p16) and CDKN1A (p21), measured after prolonged Ang II exposure. These outcomes were considered exploratory because they extend the mechanistic framework toward a stress-aging phenotype but were not used as the principal basis for defining cytoprotection.

All outcomes were predefined within this functional-to-mechanistic framework before downstream analyses were performed.

### 3.2. Cell Culture and Experimental Design

Human AC16 cardiomyocytes were obtained from the American Type Culture Collection and maintained in Dulbecco’s modified Eagle medium/F12 supplemented with 10% fetal bovine serum and 1% penicillin-streptomycin under standard humidified culture conditions (37 °C, 5% CO_2_). Cells used in all experiments were within a defined low-passage range (e.g., passages 4 - 15) to minimize passage-related phenotypic drift. For all assays, cells were seeded at densities appropriate to the experimental format and allowed to attach overnight before treatment. To model Ang II-induced cardiomyocyte injury, cells were pretreated with metformin for 2 h before Ang II exposure; metformin then remained present throughout the stimulation period. Unless otherwise specified, Ang II was used at 1.0 μM for mechanistic and functional experiments, based on preliminary dose-selection studies showing a reproducible intermediate injury window at 24 h, whereas 2.0 μM produced more severe cytotoxicity. Metformin was initially screened over a wider concentration range, and 0.5 and 1.0 mM were selected as working concentrations because they were well tolerated in AC16 cells while providing concentration-dependent protection against Ang II-induced loss of viability.

For dose-response viability experiments, the treatment groups were as follows: untreated control, Ang II alone, metformin 0.5 mM alone, metformin 1.0 mM alone, Ang II plus metformin 0.5 mM, and Ang II plus metformin 1.0 mM. For downstream mechanistic assays, the principal experimental groups were untreated control, Ang II alone, Ang II plus metformin 0.5 mM, and Ang II plus metformin 1.0 mM, with metformin-alone groups included where appropriate for calibration or control comparisons. The exposure duration was 24 h for cell viability, wound-healing, inflammatory gene expression, oxidative stress, nuclear/cytoplasmic fractionation, intracellular ROS measurement, and apoptosis-related protein analysis, whereas senescence-associated marker analysis was performed after 48 h exposure. All treatment groups within a given assay were processed concurrently under identical experimental conditions.

### 3.3. Cell Line Authentication and Quality Control

AC16 cardiomyocytes were obtained from the American Type Culture Collection and maintained according to the supplier’s recommendations. Cells were used within a limited passage range to minimize phenotypic drift. Cells were routinely monitored for morphology and contamination throughout the study.

### 3.4. Reagents and Pharmacological Treatments

Angiotensin II acetate salt (Cat. No. A9525, Sigma-Aldrich) and metformin hydrochloride (Cat. No. M8507, Sigma-Aldrich) were used in this study. Stock solutions were prepared in sterile distilled water and diluted in culture medium immediately before use to the required working concentrations. For all exposure experiments, treatment media were freshly prepared on the day of use. Metformin pretreatment was performed for 2 h before Ang II challenge, and metformin remained present throughout the subsequent Ang II exposure period until the designated endpoint.

### 3.5. Cell Viability Assay

**Figure 1.**
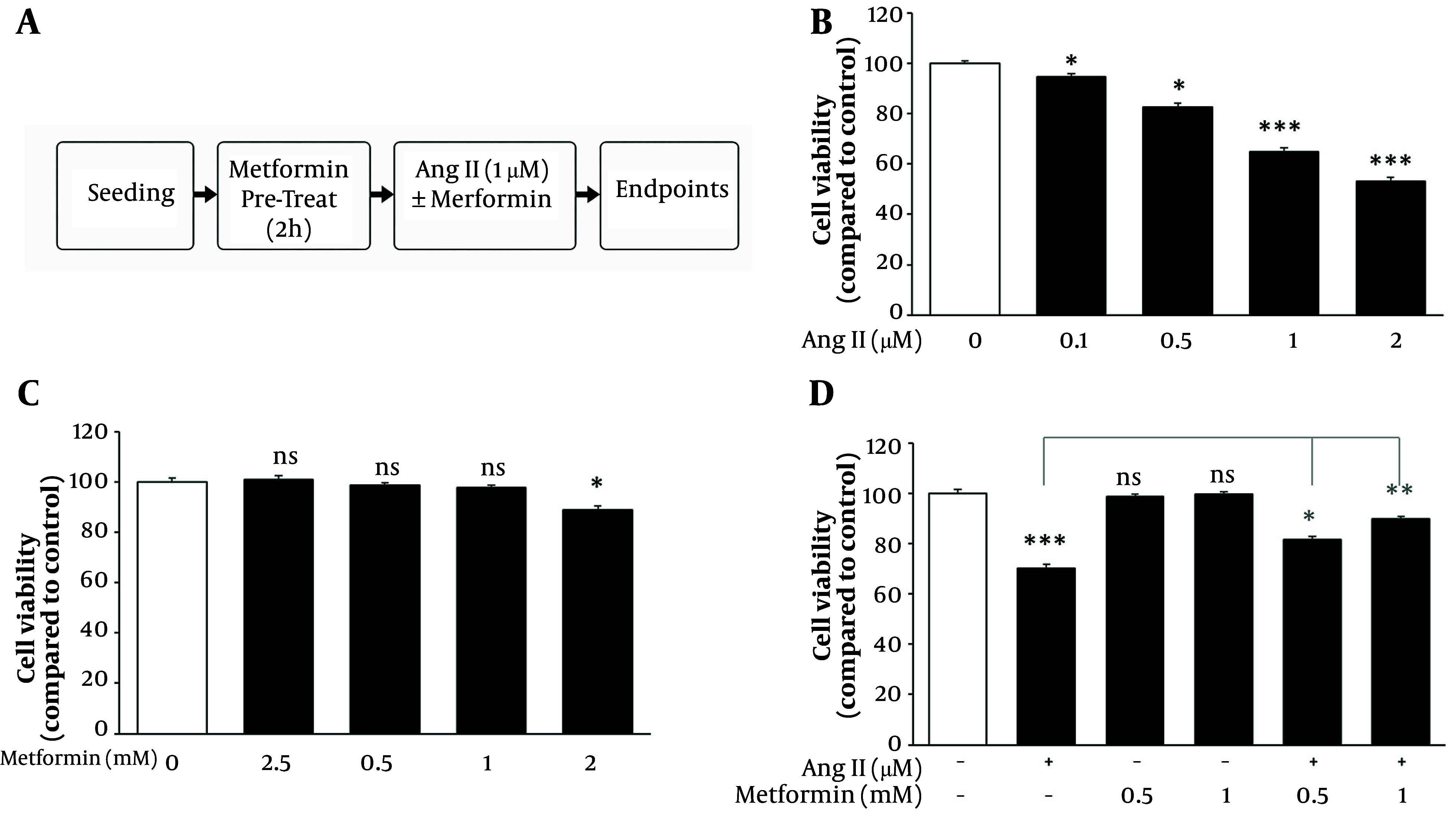
Ang II injury calibration in AC16 cardiomyocytes and protection by metformin: (A) Study workflow: AC16 cells were seeded, allowed to attach overnight, pretreated with metformin for 2 h, and then challenged with angiotensin II (Ang II) while metformin exposure was maintained until sample collection; (B) Dose-response to Ang II: Cell metabolic activity/viability was measured by MTT after 24 h exposure to 0.1 - 2.0 μM Ang II; (C) Metformin tolerability: AC16 cells were incubated for 24 h with metformin (0.25 - 2.0 mM) and assessed by MTT to define usable, non-injurious concentrations; (D) Rescue assay: Cells were pretreated with metformin (0.5 or 1.0 mM) for 2 h and then exposed to Ang II (1.0 μM) under continued metformin treatment for 24 h before viability assessment. Values are shown as mean ± SD, derived from three independent experiments, each performed with triplicate wells. Unless otherwise specified, n = 3 independent biological experiments, and triplicate wells within each experiment were treated as technical replicates and averaged before statistical analysis. Group comparisons were evaluated using one-way analysis of variance followed by Tukey’s multiple-comparison test. Statistical significance was defined as *P < 0.05, **P < 0.01, and ***P < 0.001.

AC16 cells were seeded into 96-well plates at a density of approximately 5 × 10^3^ cells per well and allowed to adhere overnight. Cells were then exposed to the indicated treatment conditions in complete medium for 24 h. For Ang II dose calibration, cells were treated with 0.1, 0.5, 1.0, or 2.0 μM Ang II. For metformin tolerability screening, cells were treated with metformin over the tested concentration range shown in Figure 1C. For rescue experiments, cells were pretreated with metformin for 2 h and then challenged with Ang II (1.0 μM) under continued metformin exposure for 24 h. Cell viability was assessed using the MTT assay (Cat. No. M5655, Sigma-Aldrich) according to the manufacturer’s instructions. Absorbance was measured at the appropriate wavelength, and viability was expressed as a percentage of the untreated control group (17, 18). Each condition was tested in triplicate wells per experiment, and the experiment was independently repeated at least three times.

### 3.6. Scratch Wound-Healing Assay

AC16 cells were seeded in 6-well plates at a density sufficient to achieve a near-confluent monolayer by the following day, approximately 3 × 10^5^ to 5 × 10^5^ cells per well, depending on the growth rate. After overnight attachment, a uniform linear scratch was created in each well using a sterile 200-μL pipette tip. Detached cells were removed by gentle washing with phosphate-buffered saline, and the wells were then replenished with treatment medium. To minimize the contribution of proliferation to wound closure, the scratch assay was performed under proliferation-restricted conditions using reduced-serum medium during the observation period. Cells were pretreated with metformin for 2 h, followed by Ang II exposure under continued metformin treatment, and images were acquired immediately after scratching (0 h) and at 24 h using the same microscope settings and marked imaging positions.

For each well, multiple non-overlapping fields along the scratch were captured in a standardized manner, avoiding irregular edges and detached-cell clusters. Wound area or scratch width was quantified using ImageJ by an investigator applying identical analysis settings across all groups. Wound closure was calculated as the percentage reduction in wound area relative to the 0 h image. All treatment groups were analyzed concurrently, and at least three independent biological experiments were performed.

### 3.7. RNA Extraction and Quantitative Real-Time PCR

Total RNA was isolated using TRIzol reagent (Cat. No. 15596026, Thermo Fisher Scientific) according to the manufacturer’s protocol. RNA concentration and purity were determined spectrophotometrically, and cDNA was synthesized using a cDNA Reverse Transcription Kit. Quantitative real-time PCR was performed using gene-specific primers under standard cycling conditions. Transcript levels of TNFA, IL6, IL1B, CDKN2A (p16), CDKN1A (p21), NFE2L2 (Nrf2), HMOX1 (HO-1), and NQO1 were normalized to GAPDH and calculated using the 2^−ΔΔCt^ method. For inflammatory and oxidative stress genes, cells were treated for 24 h; for senescence-associated markers, cells were treated for 48 h. Each condition was analyzed using biological triplicates, and the full experiment was independently repeated at least three times. Cells were seeded at a density appropriate for 6-well plate RNA harvest and exposed in complete medium for the assay-specific treatment durations described above.

### 3.8. Western Blot Analysis

Total protein was extracted using radioimmunoprecipitation assay lysis buffer supplemented with protease inhibitors, followed by centrifugation at 12,000 × g for 15 min at 4 °C to remove cellular debris, as described previously (19). Equal amounts of protein were separated by sodium dodecyl sulfate-polyacrylamide gel electrophoresis, transferred to polyvinylidene fluoride membranes, blocked, and incubated with primary antibodies against Nrf2 (Cell Signaling Technology, Danvers, MA, USA; Cat. No. 12721), HO-1 (Cat. No. 70081), NQO1 (Cat. No. 62262), NF-κB p65 (Cat. No. 8242), phospho-NF-κB p65 (Ser536) (Cat. No. 3033), IκBα (Cat. No. 4812), BAX (Cat. No. 2772), BCL-2 (Cat. No. 4223), cleaved caspase-3 (Asp175) (Cat. No. 9664), cleaved PARP (Asp214) (Cat. No. 9541), and GAPDH (Cat. No. 5174), followed by horseradish peroxidase-conjugated secondary antibodies. Protein bands were visualized using enhanced chemiluminescence and quantified by densitometric analysis. Whole-cell lysate protein signals were normalized to GAPDH. All treatment groups were run in parallel within each experiment, and representative blots were derived from experiments independently repeated at least three times. Cells were seeded in multiwell plates or dishes to ensure adequate protein yield at the time of harvest and were treated in complete medium for 24 h unless otherwise specified.

### 3.9. Nuclear and Cytoplasmic Fractionation

Nuclear and cytoplasmic fractions were isolated using a differential lysis-based subcellular fractionation protocol with sequential extraction buffers to separately recover cytoplasmic and nuclear proteins, as described previously (19), to evaluate the nuclear and cytoplasmic distribution of relevant signaling proteins. Fraction purity was confirmed using compartment-specific marker proteins, including GAPDH for the cytoplasmic fraction and lamin B1 (Cell Signaling Technology, Danvers, MA, USA; Cat. No. 13435) for the nuclear fraction. Treatments were applied for 24 h, and all experimental groups were processed simultaneously within each replicate experiment. Fractionation was performed after 24 h of treatment using matched parallel cultures processed concurrently across all groups. Nuclear protein signals were normalized to lamin B1 before comparison across treatment conditions.

### 3.10. Intracellular ROS Analysis

Intracellular ROS generation was assessed using the DCFDA Cellular ROS Detection Assay Kit (Cat. No. ab113851, Abcam) according to the manufacturer’s protocol (20). After treatment, cells were incubated with DCFDA, and fluorescence intensity was measured under identical acquisition settings across groups. Reactive oxygen species levels were quantified relative to the control group. Each condition was examined in triplicate and independently repeated at least three times.

### 3.11. Assay-Specific Treatment Allocation

The treatment structure used in this study was as follows: 1) dose-calibration MTT assay: control, Ang II alone, metformin 0.5 mM alone, metformin 1.0 mM alone, Ang II plus metformin 0.5 mM, and Ang II plus metformin 1.0 mM; 2) wound-healing assay: control, Ang II alone, and Ang II plus metformin; 3) quantitative PCR analysis of inflammatory and antioxidant-response genes: control, Ang II alone, and Ang II plus metformin; 4) quantitative PCR analysis of senescence markers: control, Ang II alone, and Ang II plus metformin after 48 h; 5) Western blotting and fractionation assays: control, Ang II alone, and Ang II plus metformin; and 6) ROS assay: control, Ang II alone, and Ang II plus metformin. All groups were run concurrently within each assay.

### 3.12. Statistical Analysis

Data are presented as mean ± SD. Unless otherwise specified, results for each assay were derived from at least three independent biological experiments performed on separate occasions. Triplicate measurements within each experiment, where applicable, were treated as technical replicates and averaged before statistical comparison. Statistical comparisons among multiple groups were performed using one-way analysis of variance followed by Tukey’s post hoc test. Given the small sample size typical of independent cell-culture experiments, data were examined for obvious distributional irregularities before parametric analysis, and all treatment groups within each assay were processed under matched conditions. For scratch wound assays, multiple image fields were quantified per condition and summarized within each independent experiment before analysis. For quantitative PCR and densitometric immunoblot analyses, values from each independent experiment were used for group-wise comparison. A P value < 0.05 was considered statistically significant. Statistical significance in graphs and figure annotations was denoted as follows: *P < 0.05, **P < 0.01, and ***P < 0.001.

## 4. Results

The results are presented according to a prespecified endpoint hierarchy, beginning with the primary functional efficacy outcomes of cell viability and repair competence, followed by secondary mechanistic analyses of inflammatory signaling, oxidative stress, antioxidant pathway activation, and apoptosis, and finally an exploratory assessment of senescence-associated markers.

### 4.1. Angiotensin II Reduces AC16 Viability in a Dose-Responsive Manner, and Metformin Confers Cytoprotection

AC16 cardiomyocytes were seeded under standardized conditions, allowed to attach overnight, and pretreated with metformin for 2 h before Ang II exposure; metformin was maintained throughout the subsequent treatment period until each assay endpoint (Figure 1A). Angiotensin II reduced MTT viability in a concentration-dependent manner at 24 h (Figure 1B). Relative to the control group (100.0 ± 1.1%), viability decreased to 94.7 ± 1.1% at 0.1 µM Ang II, 82.5 ± 1.6% at 0.5 µM, 64.9 ± 1.6% at 1.0 µM, and 53.0 ± 1.6% at 2.0 µM. Low-dose Ang II (0.1 and 0.5 µM) caused modest but statistically significant reductions versus control (P < 0.05), whereas 1.0 µM produced a reproducible intermediate injury with an approximately 35.1% loss of viability (P < 0.001). Increasing Ang II to 2.0 µM intensified cytotoxicity to an approximately 47.0% reduction relative to control (P < 0.001), suggesting a degree of injury that could confound the interpretation of pathway-specific protective effects. Therefore, 1.0 µM was selected for subsequent mechanistic experiments. Metformin dose screening demonstrated that 0.25 - 1.0 mM was well tolerated without measurable toxicity, with viability values of 98.7 ± 1.0% at 0.25 mM, 101.0 ± 1.5% at 0.5 mM, and 97.7 ± 1.0% at 1.0 mM (P > 0.05 vs control), whereas 2.0 mM reduced viability to 88.9 ± 1.5% (P < 0.05) (Figure 1C). Accordingly, 0.5 mM (Met-L) and 1.0 mM (Met-H) were used as working concentrations because they combined acceptable cellular tolerance with evidence of biological protection in the Ang II rescue setting. Under rescue conditions, Ang II (1.0 µM) significantly decreased viability from 100.0 ± 1.5% in control cells to 70.1 ± 1.5% (P < 0.001), whereas metformin alone maintained baseline viability (98.7 ± 1.0% for Met-L and 99.7 ± 1.0% for Met-H). Co-treatment with metformin mitigated Ang II injury in a dose-dependent manner, restoring viability to 81.7 ± 1.0% with Ang II plus Met-L and 89.7 ± 1.0% with Ang II plus Met-H. Thus, Met-L provided partial protection (P < 0.05 vs Ang II), whereas Met-H produced stronger recovery (P < 0.001 vs Ang II), bringing viability closer to the control range (Figure 1D). These findings established 1.0 µM Ang II as the injury-inducing condition and 0.5/1.0 mM metformin as the principal intervention doses for subsequent functional and mechanistic assays.

### 4.2. Angiotensin II Compromises Scratch Repair Capacity, and Metformin Restores Wound Closure

**Figure 2.**
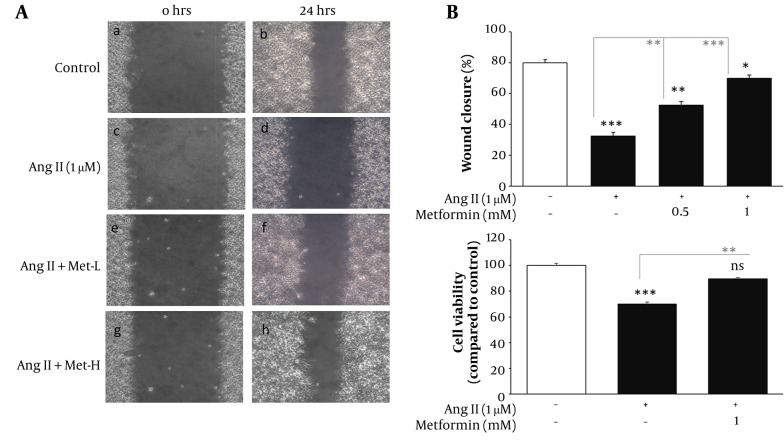
Ang II impairs scratch repair behavior and metformin restores wound closure: (A) Representative images illustrating scratch width at baseline (0 h) and after 24 h across treatment groups (control, Ang II, Ang II plus Met-L, and Ang II plus Met-H); (B) Wound closure was quantified as percent closure relative to the baseline scratch area using ImageJ-based analysis of standardized fields captured from the same wound regions at 0 h and 24 h. The assay was performed under proliferation-restricted conditions to minimize the contribution of cell proliferation to wound closure; (C) To account for viability-related effects on closure, an MTT readout was obtained at 24 h from the corresponding treatment conditions used in the scratch assay. Data are reported as mean ± SD from three independent biological repeats, with multiple fields analyzed per well under identical image acquisition settings. Unless otherwise specified, n = 3 independent biological experiments. For scratch analysis, multiple standardized image fields were quantified per condition and averaged to generate one value per independent experiment before statistical testing. Statistical testing was performed using one-way analysis of variance. Statistical significance was defined as *P < 0.05, **P < 0.01, and ***P < 0.001.

Having established a reproducible Ang II injury window and a protective metformin dose range, we next examined whether this protection extended to functional repair behavior. To evaluate functional repair, scratch assays were performed in AC16 monolayers under standardized conditions in which wounds were created in near-confluent cultures, debris was removed after scratching, and closure was monitored over a defined 24 h interval using matched imaging fields (Figure 2A). Compared with the control, Ang II markedly delayed wound closure over the 24 h observation period, consistent with impaired repair behavior. Quantitative analysis confirmed a significant reduction in closure, with control cultures showing 80.0 ± 2.0% wound closure compared with 32.3 ± 2.5% in Ang II-treated cells (P < 0.001) (Figure 2B). Metformin improved closure in a concentration-dependent manner, increasing wound closure to 52.3 ± 2.5% with Met-L (P < 0.01 vs Ang II) and to 70.0 ± 2.0% with Met-H (P < 0.001 vs Ang II), the latter approaching the control range. These findings indicate that metformin substantially restores repair-related behavior under Ang II-induced stress conditions.

Because scratch closure can be influenced by treatment-related cytotoxicity, wound measurements were interpreted alongside viability data obtained in the corresponding experimental context. Accordingly, viability was assessed under matched conditions (control, Ang II, and Ang II plus Met-H; Figure 2C). Control cells maintained viability at 100.0 ± 1.5%, whereas Ang II reduced viability to 70.1 ± 1.5% (P < 0.001 vs control). Metformin significantly improved viability to 89.7 ± 1.0% (P < 0.01 vs Ang II), indicating partial recovery of cell survival. The concordance between improved survival and enhanced wound closure supports a genuine restoration of repair competence under Ang II stress rather than an artifact driven by differential cell loss. Together, these findings demonstrate that metformin not only attenuates Ang II-induced cytotoxicity but also functionally improves wound-closure behavior in AC16 cardiomyocytes over the same observation period.

### 4.3. Metformin Suppresses Ang II-Driven Inflammatory Signaling and Reduces Senescence-Associated Gene Induction

**Figure 3.**
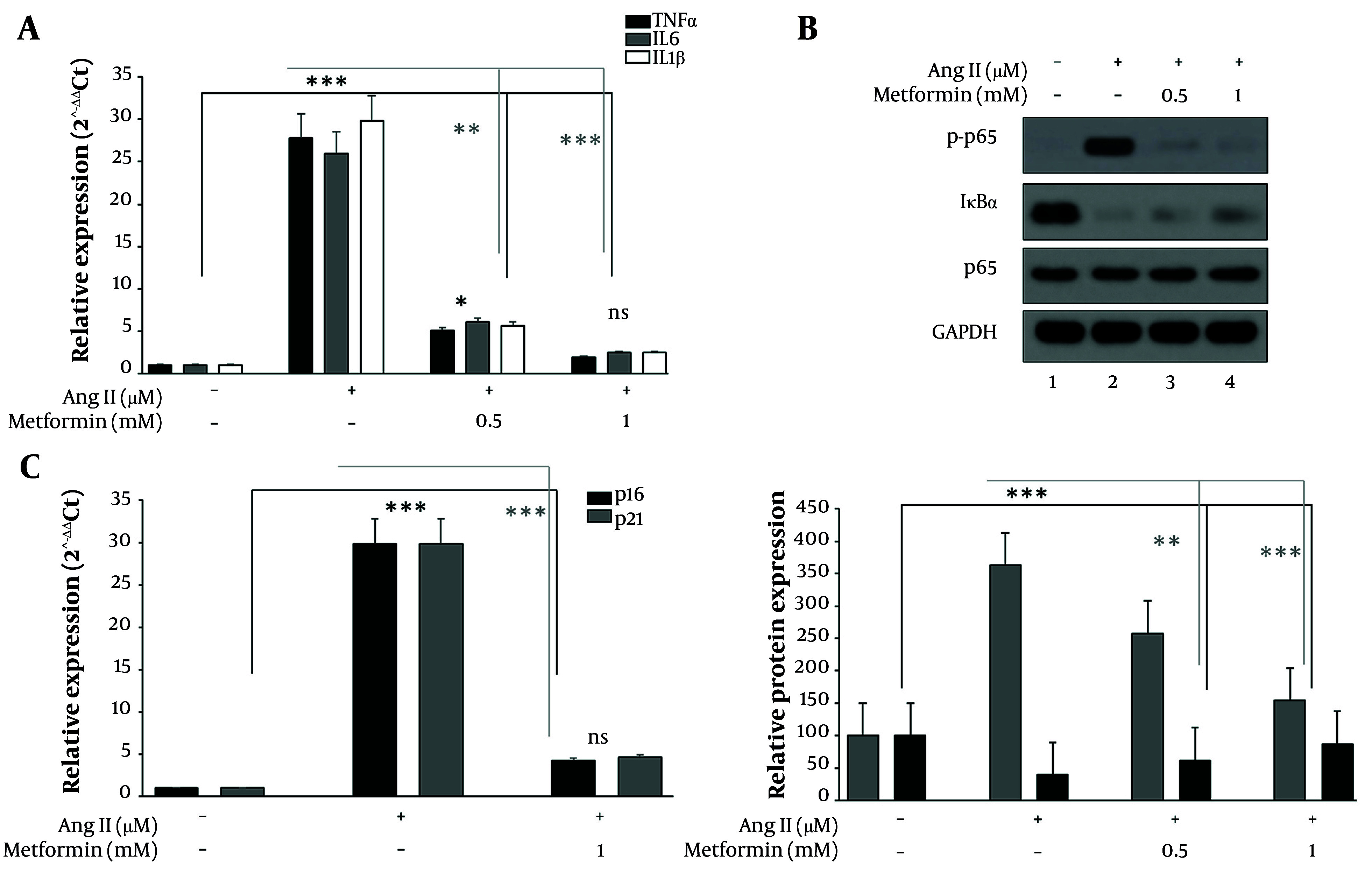
Metformin attenuates Ang II-linked inflammatory transcription, NF-κB signaling, and senescence marker induction: (A) RT-qPCR quantification of TNFA, IL6, and IL1B transcripts after 24 h Ang II exposure in the absence or presence of metformin; (B) Immunoblot assessment of NF-κB pathway status after 24 h treatment, including phosphorylated p65, total p65, and IκBα in whole-cell lysates; (C) RT-qPCR measurement of CDKN2A (p16) and CDKN1A (p21) after 48 h exposure to Ang II with or without metformin. Where applicable, immunoblot signals were quantified by densitometry and normalized to GAPDH. Results are presented as mean ± SD from three independent experiments. Unless otherwise specified, n = 3 independent biological experiments. For RT-qPCR analyses, technical reaction replicates were averaged within each biological replicate before analysis. For immunoblot quantification, densitometric values were derived from independent lysate preparations. Statistical comparisons were performed using one-way analysis of variance followed by Tukey’s multiple-comparison test. Statistical significance was defined as *P < 0.05, **P < 0.01, and ***P < 0.001.

Angiotensin II stimulation for 24 h robustly increased the mRNA expression of senescence-associated secretory phenotype-associated inflammatory cytokines TNFA, IL6, and IL1B (Figure 3A). Relative to the normalized control value of 1.00 ± 0.06, TNFA increased to 27.86 ± 2.79-fold, IL6 to 29.86 ± 2.99-fold, and IL1B to 29.86 ± 2.99-fold after Ang II exposure (P < 0.001 vs control for all). Metformin attenuated this inflammatory transcriptional response in a dose-dependent manner. With Met-L, TNFA, IL6, and IL1B were reduced to 5.04 ± 0.40-fold, 5.28 ± 0.42-fold, and 5.66 ± 0.45-fold, respectively (P < 0.01 vs Ang II), whereas Met-H further reduced these values to 1.91 ± 0.11-fold, 1.87 ± 0.11-fold, and 1.85 ± 0.15-fold, respectively (P < 0.001 vs Ang II), with expression trending toward baseline. Consistent with cytokine induction, Ang II activated NF-κB signaling (Figure 3B), as evidenced by a marked increase in phosphorylated p65 and depletion of IκBα, whereas total p65 remained essentially unchanged. Specifically, p-p65 increased from 100.0 ± 4.6% of control to 375.8 ± 13.1% with Ang II, whereas total p65 changed only minimally (103.4 ± 1.5% of control). In parallel, IκBα decreased from 100.0 ± 2.0% of control to 39.6 ± 1.7% with Ang II (P < 0.001 vs control). Metformin reduced NF-κB pathway activation in a concentration-dependent manner, lowering p-p65 to 258.0 ± 6.0% with Met-L and 147.4 ± 8.6% with Met-H, while restoring IκBα to 62.0 ± 2.8% and 87.5 ± 3.3%, respectively (P < 0.001 vs Ang II for both readouts). To assess more sustained stress programming, senescence-associated markers were measured after 48 h Ang II exposure (Figure 3C). Angiotensin II markedly increased CDKN2A (p16) and CDKN1A (p21), with both transcripts rising from 1.00 ± 0.05-fold in control cells to 29.86 ± 2.99-fold (P < 0.001 vs control). Metformin significantly suppressed this induction, reducing p16 to 4.29 ± 0.30-fold and p21 to 4.59 ± 0.32-fold (P < 0.001 vs Ang II), indicating attenuation of a senescence-like transcriptional shift under prolonged Ang II challenge.

### 4.4. Metformin Activates the Nrf2 Antioxidant Axis, Reduces ROS Accumulation, and Limits Apoptotic Signaling

**Figure 4.**
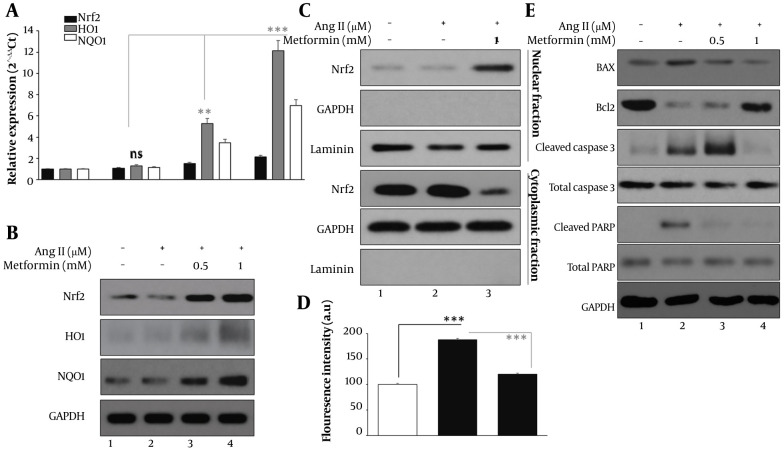
Metformin strengthens the Nrf2 antioxidant program, limits ROS accumulation, and reduces apoptosis-related protein activation: (A) RT-qPCR analysis of NFE2L2 (Nrf2), HMOX1 (HO-1), and NQO1 mRNAs following 24 h treatment; (B) Whole-lysate immunoblotting for Nrf2, HO-1, and NQO1 protein expression; (C) Subcellular fractionation with immunoblot detection to determine Nrf2 enrichment in the nuclear compartment; (D) Intracellular ROS was quantified using DCFDA-based fluorescence; (E) Immunoblot profiling of apoptotic signaling proteins (BAX, BCL-2, cleaved caspase-3, and cleaved PARP). Whole-lysate proteins were normalized to GAPDH, and nuclear fractions were normalized to lamin B. Fluorescence values are expressed relative to the control condition. Data represent mean ± SD from three independent experiments. Unless otherwise specified, n = 3 independent biological experiments. For RT-qPCR analyses, technical reaction replicates were averaged within each biological replicate before analysis. For immunoblot quantification, densitometric values were derived from independent lysate preparations, and ROS measurements were summarized from independent experiments under matched acquisition settings. Statistical comparisons were performed using one-way analysis of variance followed by Tukey’s multiple-comparison test. Statistical significance was defined as *P < 0.05, **P < 0.01, and ***P < 0.001.

Antioxidant-response genes were assessed after 24 h treatment (Figure 4A). Angiotensin II alone produced only minimal induction of NFE2L2 (Nrf2), which changed slightly from 1.00 ± 0.06-fold in control cells to 1.07 ± 0.08-fold. In contrast, metformin enhanced antioxidant gene expression in a dose-responsive manner, with NFE2L2 increasing to 1.52 ± 0.14-fold in the Ang II plus Met-L group and 2.14 ± 0.17-fold in the Ang II plus Met-H group. A stronger effect was observed for the downstream antioxidant targets. HMOX1 (HO-1) remained near baseline with Ang II alone (1.15 ± 0.08-fold) but increased to 2.83 ± 0.20-fold with Met-L and 4.29 ± 0.30-fold with Met-H. Similarly, NQO1 showed only a modest response to Ang II (1.23 ± 0.09-fold) but rose to 2.64 ± 0.18-fold and 3.81 ± 0.27-fold in the Met-L and Met-H groups, respectively. These findings indicate that metformin activates the Nrf2-linked antioxidant transcriptional program under Ang II stress.

Immunoblotting corroborated activation of this antioxidant program (Figure 4B), showing increased Nrf2, HO-1, and NQO1 protein abundance in metformin-treated cells compared with Ang II alone. The effect was concentration-dependent, with the higher metformin dose producing the strongest restoration of antioxidant protein expression, consistent with the transcriptional data.

Subcellular fractionation further supported activation of Nrf2-associated antioxidant signaling (Figure 4C). Angiotensin II slightly reduced nuclear Nrf2 relative to the control, whereas metformin increased nuclear Nrf2 enrichment, indicating enhanced translocation of Nrf2 to the nucleus under protective treatment conditions. This pattern is consistent with functional activation of the antioxidant-response pathway.

Changes in apoptosis-related proteins also supported a protective effect of metformin (Figure 4D). Angiotensin II shifted AC16 cells toward a pro-apoptotic state, increasing the BAX/BCL-2 ratio from 1.00 in control cells to 2.98, while also elevating cleaved caspase-3 from 100% to 230% of control and cleaved PARP from 100% to 212% of control. Metformin counterregulated these changes in a dose-dependent manner. In the Ang II plus Met-L group, the BAX/BCL-2 ratio decreased to 1.74, while cleaved caspase-3 and cleaved PARP declined to 165% and 149% of control, respectively. The higher metformin dose produced stronger normalization, reducing the BAX/BCL-2 ratio to 1.19, cleaved caspase-3 to 120% of control, and cleaved PARP to 112% of control, indicating substantial suppression of Ang II-associated apoptotic signaling.

Representative immunoblots for these apoptosis-associated proteins are shown in Figure 4E, illustrating that Ang II increased pro-apoptotic signaling, whereas metformin restored a more survival-favoring protein profile. In particular, the higher metformin dose was associated with lower BAX, cleaved caspase-3, and cleaved PARP, together with relative preservation of BCL-2, consistent with the densitometric summary.

Consistent with these antioxidant and anti-apoptotic effects, intracellular ROS measurements using DCFDA showed that Ang II markedly increased oxidative stress. Relative fluorescence rose from 100.0 ± 2.0 in control cells to 187.7 ± 2.5 with Ang II. Metformin significantly reduced ROS accumulation, lowering fluorescence to 120.0 ± 2.0 in the Ang II plus Met-H group. Overall, these findings indicate that metformin enhances Nrf2-associated antioxidant defenses, reduces oxidative burden, and limits downstream apoptosis-related signaling in Ang II-stressed AC16 cardiomyocytes.

### 4.5. Summary Schematic Integrates Dual Pathway Control by Metformin

**Figure 5.**
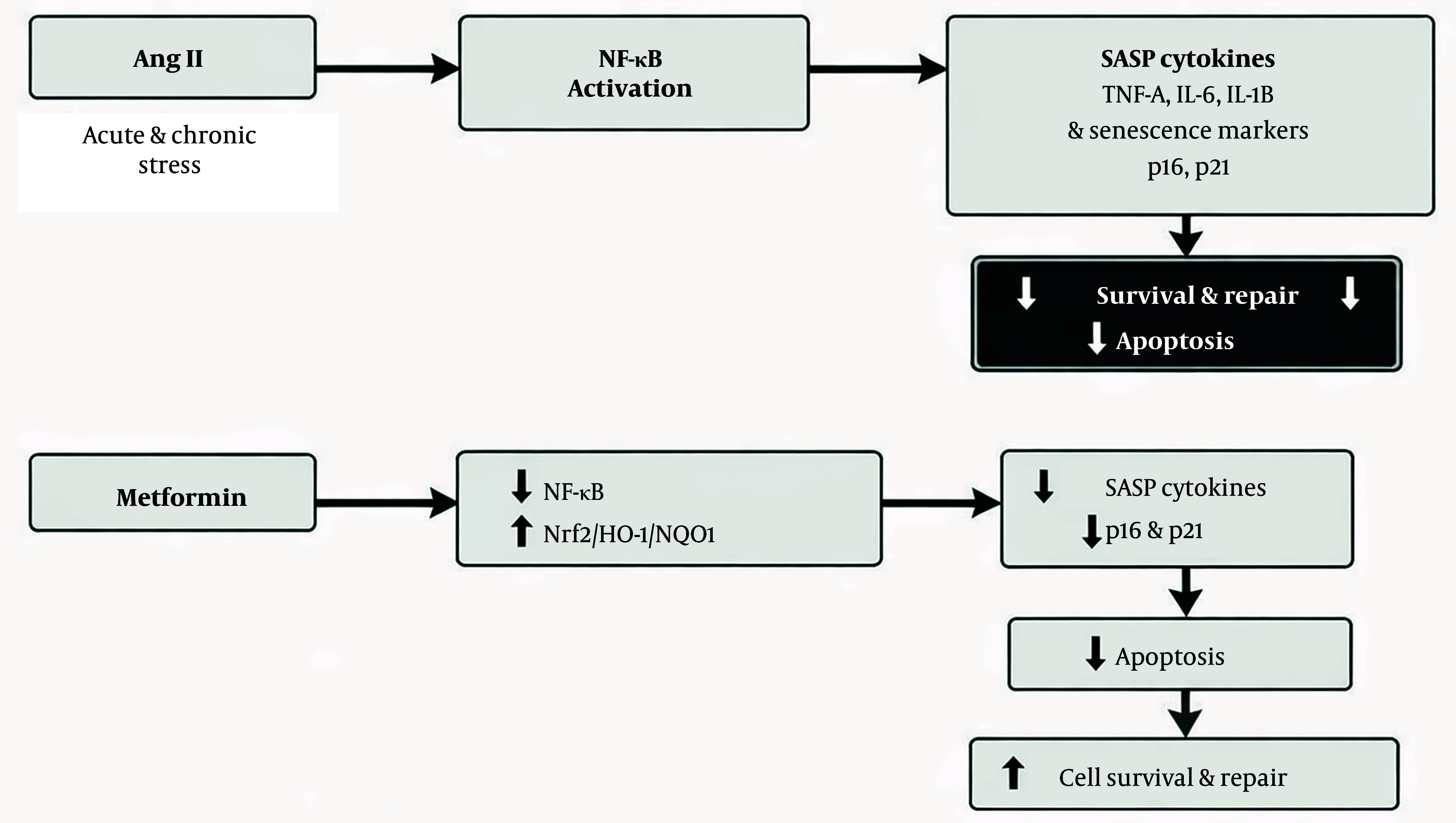
Proposed mechanistic model of metformin-mediated protection against Ang II-induced cardiomyocyte stress. Schematic illustration integrating experimental findings demonstrating Ang II-induced activation of NF-κB signaling, inflammaging-associated cytokine production, senescence marker induction, oxidative stress accumulation, and apoptotic signaling in AC16 cardiomyocytes. Metformin was associated with attenuation of these responses, together with reduced NF-κB signaling and enhanced Nrf2-dependent antioxidant pathway activity, thereby improving cellular survival and repair capacity. This model represents a mechanistically plausible interpretation of the observed findings rather than proof of direct pathway dependence.

A consolidated model (Figure 5) summarizes the experimental findings. Ang II activates NF-κB-driven inflammatory/senescence-associated secretory phenotype signaling and elevates senescence markers while increasing ROS and apoptosis, collectively impairing survival and repair. Metformin was associated with suppression of NF-κB signaling and activation of the Nrf2-HO-1/NQO1 antioxidant program, accompanied by lower ROS levels, reduced apoptotic signaling, and improved functional resilience under Ang II stress.

## 5. Discussion

This study establishes a reproducible Ang II-induced injury model in AC16 human cardiomyocytes and demonstrates that metformin confers broad cytoprotection by restoring viability and repair capacity while attenuating inflammatory, oxidative, senescence-associated, and apoptotic stress responses. As a preclinical in vitro study, these findings provide mechanistic insight within an AC16 cardiomyocyte-like model rather than direct evidence of therapeutic effects in the aging myocardium or of cardiovascular disease in vivo. Given the continuing increase in the global cardiovascular disease burden, mechanistic pharmacology studies that clarify how widely used agents improve stress resilience remain clinically relevant (21).

Angiotensin II is a central effector of the renin-angiotensin system and a well-validated driver of maladaptive cardiac signaling, involving receptor-linked kinase activation, Ca^2+^ dysregulation, and amplification of oxidative stress (22). In our model, Ang II produced a clear dose-dependent reduction in AC16 viability, with an intermediate, experimentally useful injury window at 1.0 µM after 24 h. This approach is important because excessive cytotoxicity can mask pharmacological rescue and obscure mechanistic interpretation. The observation that Ang II impaired scratch wound closure further supports a functional repair-failure phenotype. Angiotensin II-linked oxidative stress signaling is known to contribute to cardiomyocyte dysfunction and pathological remodeling, often through NOX- and mitochondria-derived ROS and redox-sensitive transcriptional pathways (23-26).

Metformin pretreatment and continued exposure significantly attenuated Ang II-induced cytotoxicity and improved wound closure in a dose-dependent manner. This aligns with prior evidence that metformin can counter Ang II-driven stress responses through energy-stress and redox-regulatory networks (27, 28). Importantly, scratch closure can be confounded by gross cytotoxicity; therefore, our parallel viability analysis in the same treatment groups strengthens the conclusion that metformin improves repair competence under stress rather than producing an apparent closure effect driven solely by differential cell survival.

Angiotensin II strongly induced TNFA, IL6, and IL1B and activated NF-κB signaling, as reflected by increased p65 phosphorylation with reduced IκBα. Angiotensin II-induced signal transduction has been closely tied to activation of inflammatory pathways, including NF-κB, and to ROS-dependent feed-forward loops that sustain injury programs (22, 23). Metformin reversed NF-κB activation and suppressed cytokine induction, consistent with literature showing that metformin can inhibit NF-κB-mediated inflammatory responses in vascular and cardiometabolic injury models (21). The broader concept of inflammaging and senescence-associated secretory phenotype-driven amplification of tissue dysfunction provides a coherent framework for interpreting the cytokine profile observed under Ang II stress (29-31).

Under extended Ang II exposure (48 h), induction of p16 (CDKN2A) and p21 (CDKN1A) indicates engagement of a senescence-like stress response. Angiotensin II is known to induce premature senescent phenotypes through ROS-dependent mechanisms, and p21-dependent senescence has been associated with Ang II biology in the vasculature (28). The suppressive effect of metformin on p16 and p21 expression suggests that metformin may mitigate chronic stress-associated senescence-like programming, although this interpretation remains provisional in the absence of orthogonal senescence assays (32).

Our results demonstrate that metformin strongly activates antioxidant pathways, including HO-1 and NQO1 upregulation, nuclear Nrf2 enrichment, and decreased ROS levels measured using DCFDA. Nuclear Factor Erythroid 2-Related Factor 2 is a master transcriptional regulator of antioxidant and cytoprotective pathways, and Nrf2 activation is a proven approach to mitigate oxidative damage. Mechanistically, attenuation of ROS levels provides a logical link between the observed downregulation of apoptotic proteins, including BAX, cleaved caspase-3, and cleaved PARP, and the recovery of BCL-2. Angiotensin II-induced oxidative stress is closely associated with mitochondrial and caspase-mediated apoptosis in cardiovascular injury models (33). Notably, metformin’s protective profile is consistent with existing literature showing that metformin attenuates oxidative stress and apoptosis in cardiovascular settings and may activate Nrf2-related protective pathways (34).

Taken together, the results support an integrated mechanism whereby Ang II triggers coupled injury circuits, including ROS amplification and NF-κB activation, driving cytokine induction, senescence-like marker upregulation, and apoptosis, culminating in loss of survival and repair. Metformin was associated with interruption of these injury-linked circuits, alongside suppression of NF-κB/senescence-associated secretory phenotype-related signaling and enhancement of Nrf2-associated antioxidant defenses, with concomitant reductions in ROS burden and downstream apoptotic signaling. This integrated response provides a mechanistically plausible explanation for the observed recovery of viability and repair under Ang II stress, but the present data do not establish definitive pathway dependence. Accordingly, these findings should be interpreted as preclinical, cell line-based, and hypothesis-generating rather than directly translatable to aging myocardium biology or cardiovascular therapy.

This study should be interpreted within the limitations of association-based mechanistic inference. Although the observed protection by metformin was accompanied by reduced NF-κB activation, enhanced Nrf2-associated antioxidant responses, lower ROS burden, and diminished apoptotic signaling, the present experiments do not establish that these pathways are strictly required for the protective phenotype. Causal pathway dependence would require dedicated perturbation approaches, such as Nrf2 knockdown or inhibition, NF-κB-directed interference, AMPK interrogation, or rescue/interference experiments. Finally, adding orthogonal senescence assays, such as senescence-associated β-galactosidase activity and senescence-associated DNA damage markers, would strengthen the senescence-like interpretation supported here by p16/p21 and senescence-associated secretory phenotype cytokines. In addition, validation in independent human cardiomyocyte systems and in vivo models will be necessary before broader biological or translational inferences can be made.

These findings are further supported by recent studies highlighting the protective effects of metformin in stress-related dysfunction through enhancement of antioxidant defense systems, as well as the broader role of systemic inflammatory processes in cardiovascular disease progression and the growing interest in therapeutic intervention strategies targeting tissue repair, collectively reinforcing the relevance of integrated stress-response modulation in cardiomyocyte injury models (35-37).

In summary, metformin attenuated Ang II-induced injury and repair impairment in AC16 cardiomyocytes, accompanied by reduced inflammatory signaling, improved antioxidant-response profiles, lower ROS accumulation, and reduced apoptosis-associated changes. These findings support a mechanistically plausible protective model in this preclinical in vitro system. However, the study is based on a single immortalized cardiomyocyte-like cell line and should therefore be interpreted as hypothesis-generating rather than directly translatable to aging myocardium biology or cardiovascular therapy. Further validation in additional human cardiomyocyte models and in vivo systems is required.

## Data Availability

The dataset presented in the study is available on request from the corresponding author during submission or after publication.
